# 

**Published:** 1840-08

**Authors:** 


### CONTENTS

OF NO. VIII.

### ORIGINAL COMMUNICATIONS.

### ESSAYS AND CASES.

ART. I.—Observations on the Nature and Treatment of Spina Bifida. By AMASA TROWBRIDGE, M.D., Professor of Surgery in Willoughby University, Ohio. - - - - - 85

ART. II.—Remarks on Scarlet Fever as it prevailed in Calloway county, Ky. By DR. W. S. LAWRIE, of Kentucky. - 90

ART. III.—A case of Fracture of the Cranium with loss of Brain. By A. H. BUCHANAN, M.D., of Columbus, Tenn. - 96

ART. IV.—A case of Chronic Enlargement of the Spleen, with Remarks. By RICHARD R. DASHIELL, M.D., of Tennessee. - - - - - - - - - - 99

ART. V.—Observations on Milk-Sickness. By JOHN TRAVIS, M.D., of Carroll county, Tennessee. - . - - - 101

### REVIEWS.

ART. VI.—Crania Americana; or a comparative view of the Skulls of various Aboriginal nations of North and South

America; to which is prefixed an Essay on the Varieties of the Human Species. Illustrated by seventy-eight plates, and a colored map. By SAMUEL GEO. MORTON, M.D., Professor of Anatomy in the Medical Department of Pennsylvania College at Philadelphia. 105

ART. VII.—Elements of Pathological Anatomy, illustrated by numerous engravings. "In Morbis, sive acutis, sive chronicis, viget occultum, per humanas speculationes fere incomprehensible."--Baglivi. By SAMUEL D. GROSS, M.D. Late professor of General Anatomy, Physiology, and Pathological Anatomy, in the Medical Department of the Cincinnati College. Vol. II, 8vo., Boston, 1839. Marsh, Capen, Lyon & Webb and James B. Dow. - 131

ORIGINAL INTELLIGENCE.

Treatment in the Case of Forcible Removal of the Uterus. By DR. BALLARD. - - - - - - - - - - 156

Medicine in Paris—Trousseau on Iron. By DR. LINTON. - - 159

Medical properties of the Cotton-Plant. By Dr. BOUCHELLE. - - 162

